# Transdermal Optical Imaging Reveal Basal Stress via Heart Rate Variability Analysis: A Novel Methodology Comparable to Electrocardiography

**DOI:** 10.3389/fpsyg.2018.00098

**Published:** 2018-02-08

**Authors:** Jing Wei, Hong Luo, Si J. Wu, Paul P. Zheng, Genyue Fu, Kang Lee

**Affiliations:** ^1^The Affiliated Hospital of Hangzhou Normal University, Hangzhou Normal University, Hangzhou, China; ^2^Dr. Eric Jackman Institute of Child Study, University of Toronto, Toronto, ON, Canada; ^3^Department of Psychology, Hangzhou Normal University, Hangzhou, China; ^4^Department of Psychology, Zhejiang Normal University, Jinhua, China

**Keywords:** transdermal optical imaging, hemoglobin concentration, stress, basal stress, respiratory sinus arrhythmia, vagal tone, autonomic nervous system, heart rate variability

## Abstract

The present study examined the validity of a novel physiological measurement technology called transdermal optical imaging (TOI) technology at assessing basal stress. This technology conveniently, contactlessly, and remotely measures facial blood flow changes using a conventional digital video camera. We compared data from TOI against the pulse data collected from the FDA approved BIOPAC system. One hundred thirty-six healthy adults participated in the study. We found that TOI measurements of heart rate and heart rate variability (HRV), which reflects basal stress, corresponded strongly to those obtained from BIOPAC. These findings indicate that TOI technology is a viable method to monitor heart rate and HRV not only accurately but also conveniently, contactlessly, and remotely. Further, measures of HRV obtained via TOI serves as a valid index of basal stress. Potential applications of this technology in psychological research and other fields are discussed.

## Introduction

Humans encounter various stressful situations everyday at work, home, and school. Such stress when experienced at high degrees and/or for a long duration of time could lead to cardiovascular diseases, cognitive dysfunctions, and psychological disorders (Kofman et al., 2006; Pan and Li, 2007; Crowley et al., 2011). Currently, the assessment of stress relies on the analysis of psychometric (e.g., self-report questionnaires) and/or biometric (e.g., electrocardiography) data. While psychometric data can provide a glimpse into an individual’s psychological state and stress level, it is heavily dependent upon a subjective reflection of events and conditions. On the other hand, biometric data can provide an objective evaluation of physiological activity that has been demonstrated to correlate well with psychological stress (Sharpley and Gordon, 1999; Tavel, 2001). However, biometric data are often obtained using instruments which require the attachment of electrodes or sensors onto the body by trained individuals. This use of physiological measurement instruments can be inconvenient. Thus, to date, we still face difficulties in monitoring stress levels both reliably and conveniently. The present research aimed to address these difficulties directly.

Over the last half century, research has revealed that human physiological changes in response to psychological stress, such as the amplitude of respiratory sinus arrhythmia (RSA), can reflect individual stress (Porges, 1995). When individuals encounter a stressful situation where a threat is perceived, their autonomic nervous system (ANS) works to adjust the internal state of their body and react to the situation. The two branches of ANS, the sympathetic and parasympathetic nervous systems, contribute in stress reaction. The sympathetic nervous system is concerned with challenges from the external environment, triggering the fight-or-flight response in stressful situations. In contrast, the parasympathetic nervous system is concerned with returning the body to a resting state or the state of homeostasis. When an individual experiences stress, the parasympathetic nervous system struggles to maintain homeostasis (Porges, 1995). Thus, an assessment of stress can be obtained by examining the level of homeostasis.

As part of the parasympathetic nervous system, the vagus nerve plays an essential role in the regulation of homeostasis because it is responsible for signaling the heart, lungs, and digestive tract to slow down and relax. The activity of the vagus nerve, otherwise known as vagal tone, would then be indicative of the level of homeostasis within the body. If individual stress decreases, then vagal tone increases, the heart slows down, and homeostasis is maintained. If individual stress increases, then vagal tone decreases, the heart quickens, and homeostasis is disrupted. A recent review of research by Castaldo et al. (2015) showed that parasympathetic vagal activity, as determined by heart rate variability (HRV) time series computed from electrocardiography (ECG) recordings, indeed decreases reliably during sessions involving stress. In addition, irregular increase and decrease of vagal tone would indicate chronic stress.

Although vagal tone can provide insight into an individual’s stress level, the changes in vagal tone cannot be measured directly. Rather, vagal tone and corresponding information involving stress can be measured indirectly but reliably by RSA. RSA is the rhythmic increase and decrease in the beating of the heart, which occurs in the presence of breathing (Berntson et al., 1997). The heart rate increases with inhalation and decreases with exhalation. Studies have shown that a decrease in resting RSA is indicative of increased stress (Watkins et al., 1998; Friedman, 2007; Jonsson, 2007; Kemp et al., 2010; Kogan et al., 2012).

In order to obtain a measurement of RSA, variations in heartbeat must first be measured. Experimental evidence primarily relies on the use of ECG to observe HRV, analyzing the time period in milliseconds between each R-wave to obtain the R-R Interval (RRI). With information regarding the RRI, inferences can be made about stress. An increasing RRI variation indicates excitation of the vagus nerve as it works to decrease heart rate, and thus we can infer stress level to be low. A decreasing RRI variation indicates an inhibited vagus nerve, allowing heart rate to increase, and thus we can infer stress level to be high (Castaldo et al., 2015, 2016). However, assessment of RRI is not enough to determine vagal tone. The issue is that respiration is not the only contributor to variations in heart rate. There are oscillations at frequencies slower than that of respiration, such as Traube-Hering-Mayer waves, which provides information regarding the sympathetic nervous system rather than the parasympathetic nervous system and stress (Porges, 1986). Thus, data from ECG recordings must be filtered to obtain various HRV features, including measurement of RSA and in effect an estimate of vagal tone that can provide information regarding individual stress levels.

Based on the evidences of cardiovascular changes in response to stress, we have specifically developed a new imaging technology called transdermal optical imaging (TOI) to assess stress conveniently, contactlessly, and remotely. This technology uses a conventional digital camera to video record participants’ faces from a distance, analyzing facial blood flow information to obtain participants’ heart rate and HRV.

Our TOI technology is built upon a century of research that has revealed cardiovascular activities to be obtainable via analyses of blood flow changes. It is well-established that light can travel beneath the skin and re-emit due to the translucent property of the skin (Brunsting and Sheard, 1929; Edwards and Duntley, 1939; Dawson et al., 1980). Furthermore, this re-emitted light can be captured by an optical sensor, from which blood flow information can be obtained (Anderson, 1991; Stamatas et al., 2004; Demirli et al., 2007). Information regarding blood flow changes reveal cardiovascular changes given that movement of blood from the heart to the rest of the body is part of the cardiovascular system. These discoveries have lead to the development of various methodologies (e.g., laser Doppler flowmetry, photoplethysmography) that measure cardiovascular activities optically. However, similar to the utilization of electrocardiography, these methodologies require the attachment of sensors to the body, which can be inconvenient.

**FIGURE 1:**
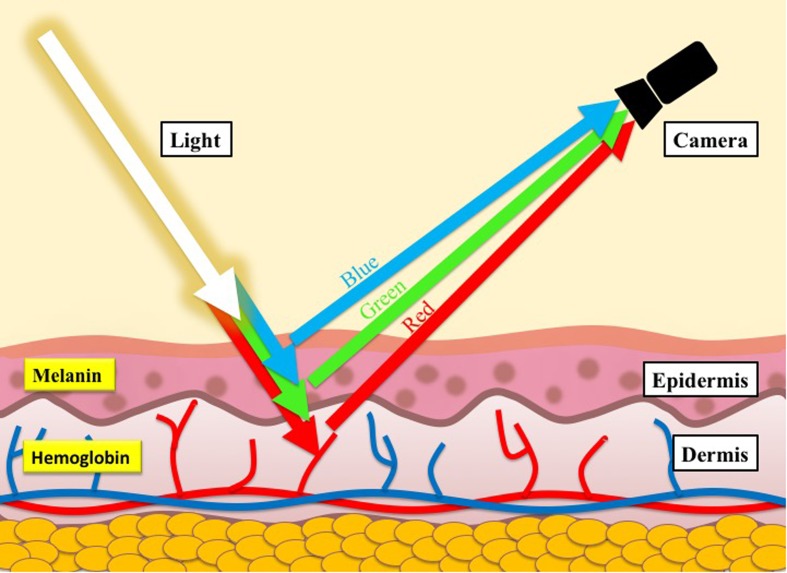
Illustration of the skin structure and the skin color model.

Transdermal optical imaging overcomes the limitations of current methodologies by utilizing a digital video camera to conveniently, contactlessly, and remotely capture video images of the face for extraction of cardiovascular changes. This is possible because re-emitted light from underneath the skin is affected by chromophores, primarily hemoglobin and melanin (Nishidate et al., 2004), which have different color signatures. Given the difference in the color signatures, we can use machine learning to separate images of hemoglobin-rich regions from melanin-rich regions, ultimately obtaining video images of hemoglobin changes under the skin (**Figure 1**; for details, see Lee and Zheng, 2016). The face is ideal for analysis of blood flow changes because it is rich in vasculature and exposed, allowing us to obtain blood flow information conveniently, contactlessly, and remotely.

In the present study, we examined the validity of TOI in measuring heart rate and HRV, which reflects individual stress. We measured participants’ cardiovascular activities while they were in a state of rest to assess their basal stress levels. We used the TOI methodology to obtain facial blood flow data reflecting heart rate, HRV, and basal stress levels. At the same time, in order to validate our TOI methodology, we compared the measurements obtained from TOI with those collected concurrently from an ECG system. We hypothesized that if there is a high positive correlation between data obtained from TOI and ECG, then cardiovascular changes as assessed by TOI should correspond with those by ECG, which were previously proven to correlate with individual stress. Thus, we would provide evidence to suggest TOI to be a valid methodology for assessing stress conveniently, contactlessly, and remotely.

## Method

### Participants

One hundred thirty-six healthy adults above 18 years of age (57 males; mean age = 24.33; *SD*: 8.62) participated in the study. Participants were given full disclosure of the research protocol before being presented with a consent form. All participants signed a written informed consent form prior to the experiment. The present study was conducted in accordance with the NIH research ethics guidelines, and was approved by Research Ethics Review Committee at the University of Toronto and Hangzhou Normal University.

### Materials

A 2-min video of animated clouds moving through the sky was presented to participants on a computer screen, using E-prime 2.0. The computer screen was placed on a table in the center of the study room.

The FDA approved BIOPAC physiological measurement system, BIOPAC MP150 (BIOPAC Systems, Inc., Goleta, CA, United States) was used to collect ECG data. Specifically, the electrocardiogram amplifier module (ECG100C) was connected to the BIOPAC system to record ECG signals. A three-lead configuration was used for collecting ECG data. The White lead was connected to SHIELD and VIN- on the ECG100C module. The Red lead was connected to SHIELD and VIN+. Finally, the Black lead was connected to GND. Alcohol swabs were used to clean participants’ skin prior to the attachment of electrodes. Disposable electrodes were placed on participants’ skin to obtain ECG data. ECG signals were displayed on a laptop, using AcqKnowledge v. 4.1 (BIOPAC Systems, Inc., Goleta, CA, United States).

**FIGURE 2:**
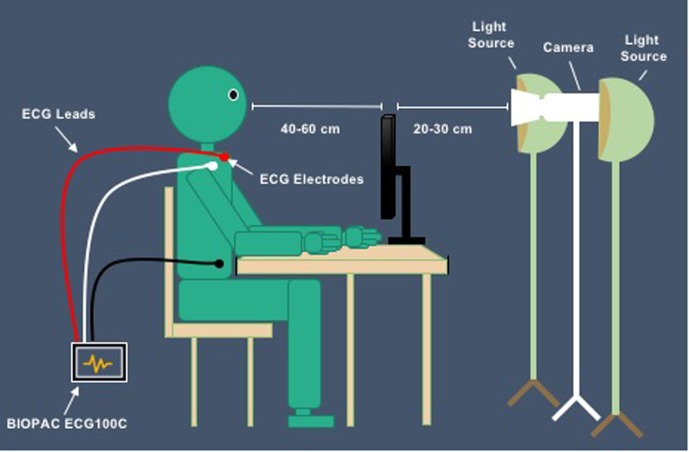
Set up of study.

A novel TOI system was constructed to obtain the transdermal image sequences as participants viewed the cloud film. A digital video camera (Canon VIXIA HF R62) was positioned on a tripod approximately 20–30 cm away from the back of the computer screen, angled to record the participants’ face at 60 frames/seconds. LED lights were positioned on both sides of the camera, uniformly illuminating the participant’s face (see **Figure 2**). The imaging system was color-calibrated to ensure successful repetitive performance with reliable and accurate color measurements.

### Procedure

Participants were tested individually, seated in front of the computer screen in a quiet room, under supervision from an experimenter. Participants were asked to sit naturally, and at a comfortable distance (about 40–60 cm) from the computer screen while the experimenter sat behind the participant, at the corner of the room, out of the camera and participants’ view. Next, participants were told that they would be presented with a relaxing film of clouds moving through the sky and they must keep their eyes on the computer screen while maintaining a neutral facial expression throughout the duration of the film.

**FIGURE 3:**
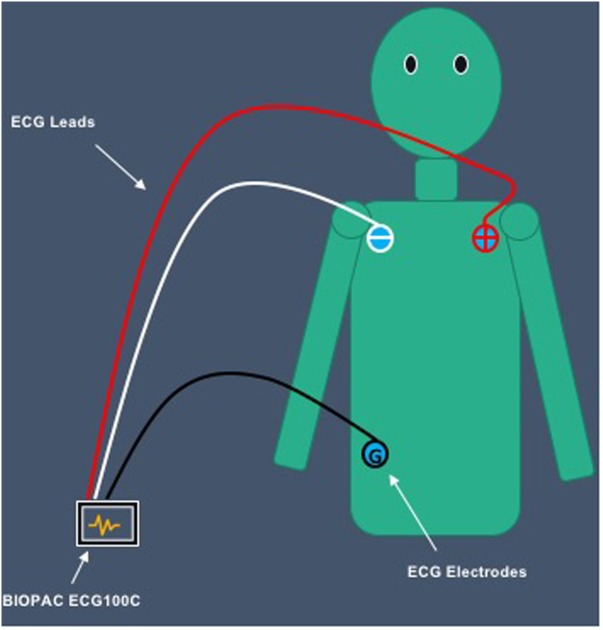
Placement of ECG electrodes on participants’ body.

Before the cloud film was presented to participants, the experimenter instructed participants to place ECG electrodes based on Einthoven’s triangle; near the right shoulder, left shoulder, and right hip. The White lead was attached to the electrode placed on participants’ right shoulder. The Red lead was attached to the electrode placed on participants’ left shoulder. Finally, the Black lead was attached to the electrode placed on participants’ right hip (see **Figure 3**).

Once a clear ECG signal was presented on the laptop with AcqKnowledge, the experimenter adjusted the digital video camera to capture participants’ face and began recording. With both TOI and BIOPAC, collecting physiological data from participants, the experimenter presented the cloud film to participants for 2 min.

### Data Analysis

#### BIOPAC ECG Analysis

Each participant’s raw ECG data was processed using MATLAB (The MathWorks, Inc.). First, we estimated the R-wave peaks and the difference in time interval between the peaks to generate the RRI and in effect the HRV. Next, we used Poincaré plot (Kamen et al., 1996) to analyze various features of HRV, specifically SD1/SD2, as research has shown this feature to be indicative of stress (Melillo et al., 2011). We plotted each individual’s RRI against the next RRI on a graph with RR(n) on the x-axis vs. RR(n+1) on the y-axis.

In order to analyze SD1/SD2, we first determined SD1, which is defined as the dispersion (standard deviation) between points in the direction perpendicular to the line of identity on the Poincaré plot. SD1 reflects the short-term variation of heart rate caused by RSA, and thus are thought to indicate the activation of the sympathetic nervous system. SD1 measurements were obtained using the following formula:

$$SD1=\frac{\sqrt{2}}{2}SD\,\;\left({RR}_{n}-{RR}_{n+1}\right)$$

Next, we determined SD2, which is defined as the dispersion (standard deviation) between points along the line of identity on the Poincaré plot. SD2 reflects the long-term variation of heart rate caused by RSA, and thus are thought to indicate the activities of the sympathetic and parasympathetic nervous system. SD2 measurements were obtained using the following formula:

$$SD2=\sqrt{2SD{\left({RR}_{n}\right)}^{2}-\frac{1}{2}SD{\left({RR}_{n}-{RR}_{n-1}\right)}^{2}}$$

Finally, we determined SD1/SD2, which is defined as the ratio of dynamic change in the HRV time series. SD1/SD2 reflects the relationship between the sympathetic and parasympathetic nervous system, which can be used as an indicator of individual stress level, with the lower ratios to indicate higher levels of stress.

#### Transdermal Optical Imaging Analysis

Transdermal optical imaging analysis is a novel imaging method that is capable of isolating hemoglobin concentration (HC) from raw human face images taken from a conventional digital camera. This analysis is based on the fact that the human facial skin is translucent (Brunsting and Sheard, 1929; Edwards and Duntley, 1939; Dawson et al., 1980). Light travels beneath the skin, and re-emits after traveling through different skin tissues. The re-emitted light may then be captured by optical cameras (Anderson, 1991; Stamatas et al., 2004; Demirli et al., 2007). The dominant chromophores affecting the re-emitted light are hemoglobin and melanin (Nishidate et al., 2004). Since hemoglobin and melanin have different color signatures, it has been found that it is possible to obtain images mainly reflecting HC under the epidermis. Capitalizing on this, TOI analysis first obtains each captured image, and then performs operations upon the image to generate a corresponding optimized HC image of a participant’s face.

Isolating HC is accomplished by analyzing bitplanes in the video sequence to determine and isolate a set of the bitplanes that provide high signal-to-noise ratio (SNR) with regard to the facial cardiovascular activities. The determination of high SNR bitplanes is made with reference to a first training set of images constituting the captured video sequence coupled with facial blood flow measurements concurrently taken with FDA approved medical instruments that measure cardiovascular activities on the face (facial blood flows with a laser Doppler machine, and blood pressure waves with a continuous cuff-based oscillatory blood pressure monitor).

With respect to bitplanes, a digital image consists of a certain number of pixels; typically referred to as a configuration of width-times-height. Each pixel has one or more channels associated with it. Each channel has a dynamic range, typically 8 bits per pixel per channel. For color videos, each image typically has three channels: Red, Green, and Blue (RGB). As such, a bitplane is a view of a single bit of an image across all pixels (i.e., a 1-bit image per bit per channel).

Using the raw images that consist of all bitplanes of all three RGB channels, signals that change over a particular time period (e.g., 120 s) on each of the pixels are extracted. Using the signals from each pixel, machine learning is employed to systematically identify bitplanes that will significantly increase the signal differentiation and bitplanes that will contribute nothing or decrease the signal differentiation. After discarding the latter, the remaining bitplane images optimally determine the blood flow. To further improve SNR, the result can be fed back to the machine learning process repeatedly until the SNR reaches an optimal asymptote. The machine learning process involves manipulating the bitplane vectors using image subtraction and addition to maximize the signal differences in all ROIs over the time period for a portion (e.g., 70, 80, 90%) of the subject data and validate on the remaining subject data. The addition or subtraction is performed in a pixel-wise manner. The resulting images thus contain information corresponding to HC in each pixel, which were then put together as video images to reflect HC changes in all parts of the face (for details, see Lee and Zheng, 2016).

**FIGURE 4:**
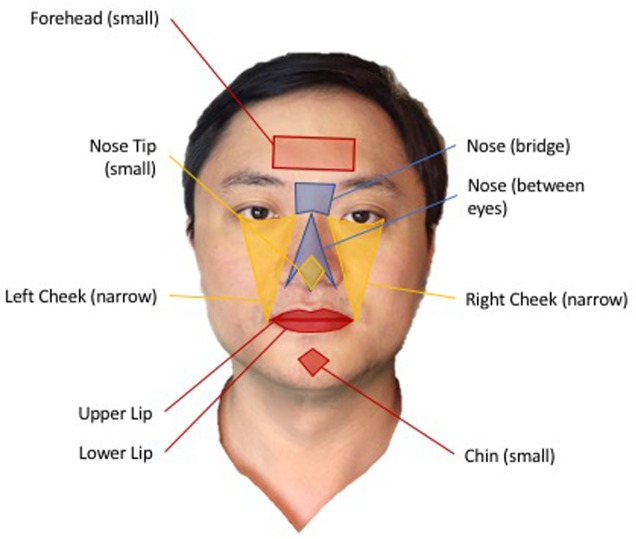
Nine regions of interests (ROIs) illustrated on the face of Paul Zheng, an author of this paper. All participants, whose image have been used for this paper, signed a written informed consent for release of their images in published articles prior to inclusion of their image in this paper.

For the present study, we divided the face into nine regions of interests (ROIs): Forehead Small, Nose Between Eyes, Nose Bridge Full, Nose Tip Small, Right Cheek Narrow, Left Cheek Narrow, Upper Lip, Lower Lip, Chin Small (**Figure 4**). We averaged the data obtained from all pixels in each ROI to further increase SNR. Next, we applied Hilbert-Huang transform to filtered ROI signal (Li et al., 2011). The transform provided us with the principle frequency component of TOI signal. Using synthesized frequency, peaks of heartbeat were reconstructed to obtain heart rate and the intervals between heartbeats (i.e., RRI) were measured.

Using the above process, each video of participant’s face was analyzed for facial blood flow information that reflects cardiovascular activities that correlate with stress. With a reconstruction of the peaks of heartbeat and a measurement for RRIs, we obtained stress in the same way that we extracted the information from data collected using the BIOPAC ECG. We plotted the RRIs on a Poincaré plot and analyzed for HRV features, specifically SD1/SD2.

### Statistical Analysis

To compare the data collected from TOI against those from the BIOPAC ECG, we used MATLAB to assess the agreement and correlation between TOI and BIOPAC measurements, specifically for measures of heart rate and SD1/SD2 (i.e., stress). We assessed agreement by constructing Bland–Altman plots for the measures of heart rate and stress obtained from TOI and BIOPAC. The Bland–Altman plot is often used to determine the differences between two measurements, with the mean difference signifying bias and the standard deviation of the differences signifying the limits of agreement. We computed 95% limits of agreement for comparison of TOI and BIOPAC measurements. We assessed correlation by calculating for the correlation coefficients between measures of heart rate and stress obtained from TOI and BIOPAC.

## Results

**FIGURE 5:**
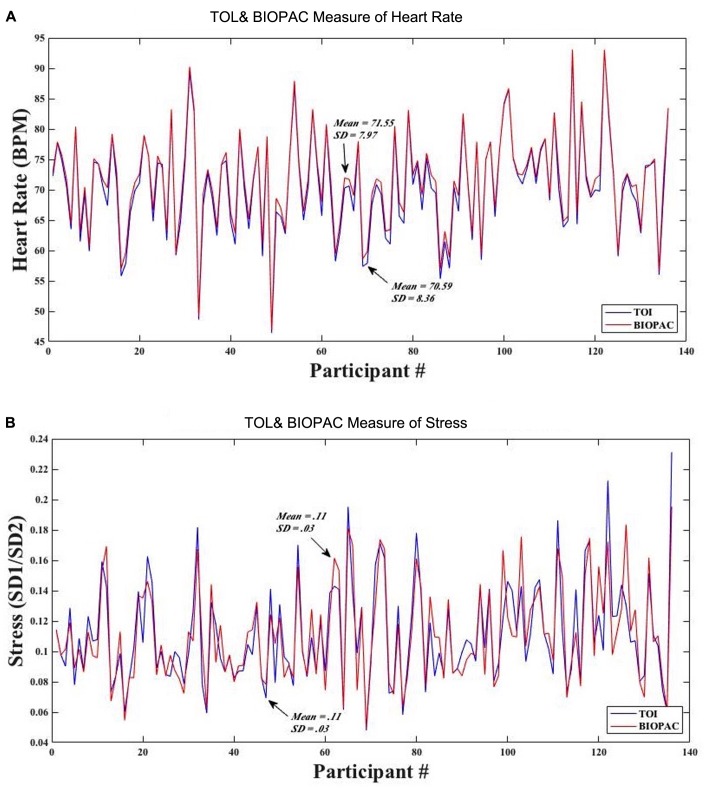
**(A)** Mean and standard deviation of participants’ heart rate obtained from TOI and BIOPAC. **(B)** Mean and standard deviation of participants’ stress from TOI and BIOPAC.

To assess the accuracy of the TOI technology, we compared measurements of heart rate and stress obtained with TOI against those obtained with the BIOPAC ECG. **Figure 5A** shows that the average (SD) of heart rate as obtained from TOI was 70.59 (8.36) beats per minute (BPM), while that obtained from BIOPAC was 71.55 (7.97) BPM. **Figure 5B** shows that the average (SD) of SD1/SD2 (i.e., stress) as obtained from TOI was 0.11 (0.03), while that obtained from BIOPAC was 0.11 (0.03).

**FIGURE 6:**
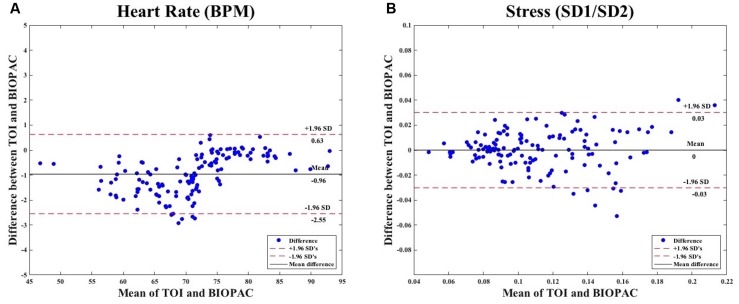
**(A)** Bland–Altman Plot comparing measures of heart rate obtained from TOI and BIOPAC. **(B)** Bland–Altman Plot comparing measures of stress obtained from TOI and BIOPAC.

We calculated for the agreement between heart rate measurements obtained from TOI and BIOPAC. We found that the agreement limits are from -2.55 to 0.63, with a bias of -0.96 (see **Figure 6A**). Next, we calculated for the agreement between stress measurements obtained from TOI and BIOPAC. We found that the agreement limits are from -0.03 to 0.03, with a bias of 0 (see **Figure 6B**).

**FIGURE 7:**
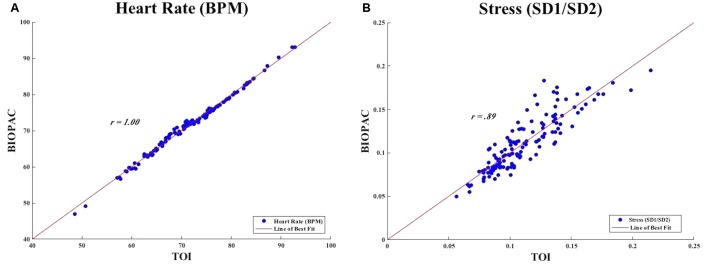
**(A)** Measures of heart rate from TOI and BIOPAC. **(B)** Measure of stress from TOI and BIOPAC.

We calculated for the correlation between heart rate measurements obtained from TOI and BIOPAC. We found that there was a positive correlation between the two instruments, *r* = 1.00. **Figure 7A** demonstrates the points of heart rate (BPM) as obtained from TOI and BIOPAC with a line of best fit drawn through the points to illustrate the positive correlation. This extremely strong, positive correlation between measurements of heart rate obtained from TOI and those obtained from the BIOPAC ECG indicated that TOI technology is able to measure heart rate as accurately as the BIOPAC ECG.

We calculated for the correlation between stress measurements obtained from TOI and BIOPAC. We found that there was a positive correlation between the measurements of stress obtained from TOI and BIOPAC, *r* = 0.89. **Figure 7B** demonstrates the points of stress (SD1/SD2) as obtained from TOI and BIOPAC with a line of best fit drawn through the points to illustrate the positive correlation. This strong, positive correlation between measurements of stress obtained from TOI and BIOPAC indicated that TOI technology is able to determine stress as accurately as the BIOPAC.

In summary, we found that there were strong, positive correlations between physiological measurements obtained from TOI and those obtained from the BIOPAC ECG. Thus, results revealed TOI to be a contactless instrument that can determine changes in human physiology, specifically heart rate and stress level, with the same amount of accuracy as existing medical instruments used for heart rate and stress assessment.

## Discussion

The present study revealed strong positive correlations between measurements of heart rate and stress as obtained from the novel TOI technology and from FDA approved BIOPAC physiological measurement system, specifically the ECG100C. We found that by applying the TOI methodology, we can use a conventional digital camera on the face to accurately determine cardiovascular changes and psychological stress conveniently, contactlessly, and remotely.

The ECG is currently the most effective instrument to be used at determining cardiovascular activities and in particular, HRV with regard to measuring individual stress. Specifically, extensive amounts of research have been utilizing HRV features computed from ECGs to indirectly derive stress levels (Sharpley and Gordon, 1999; Tavel, 2001). However, the utilization of ECG and other instruments for extraction of HRV (e.g., photoplethysmography; Giardino et al., 2002) can be inconvenient given the need to attach electrodes or sensors onto the body. In contrast, TOI conveniently utilizes a regular digital camera and machine learning algorithms to extract HRV and infer stress levels contactlessly and remotely. Our findings of high positive correlation between measurements obtained from TOI and BIOPAC ECG testifies to the validity of TOI in determining heart rate and HRV to derive stress.

Of course, while the present study’s results highlight the potential value of using TOI to measure heart rate, HRV, and to derive stress, several limitations should be acknowledged. First, the study involved a small sample of 136 adults. With such a small sample it is difficult to determine whether the strong correlation between measurements obtained from TOI and BIOPAC can be generalized to a broader population. There is a need to assess the validity of TOI against the ECG on more people. Second, the study only compared data obtained from TOI against those from ECG, but there are other methods of stress assessment such as questionnaires for self-reports of stress and photoplethysmography (PPG; Giardino et al., 2002) that has also been proven to be reliable in extracting HRV features which reflect stress. There is a need to compare data obtained from TOI with those from other reliable stress assessment methods to further validate TOI.

Third, we only analyzed non-linear features of HRV, specifically SD1/SD2, which is indicative of stress (Melillo et al., 2011), but time and frequency domain features have also been shown to reliably correlate with stress. Time domain features of HRV such as the standard deviation of an RRI (SDRR), the square root of mean squared difference of successive R-Rs (RMSSD), and the proportion of NN50 divided by total number of normal-to-normal (NN) interval that differ more than 50 ms (pNN50) have been shown to decrease under stress. In addition, frequency domain features of HRV such as high frequency power (HF), and low frequency to high frequency power ratio (LF/HF) have been shown to reliably correspond with stress such that the former decrease with stress while the latter increase with stress (Giardino et al., 2002; Shaffer et al., 2014; Castaldo et al., 2015). Thus, there is a need to conduct further analysis of HRV features obtained via TOI.

Fourth, the study only assessed participants’ stress during a 2-min resting period, which is relatively short (Smith et al., 2013; Munoz et al., 2015). Preferably, the recording duration should be longer, for example, for 5–6 min (Tharion et al., 2009; Castaldo et al., 2015). Future studies need to compare whether the short and long recording durations would produce consistent measurements. Furthermore, the present study used a relaxing video and therefore only provided us with information regarding participants’ basal stress level. Further research is needed to include both resting and stressful periods to investigate whether TOI can accurately pick up acute stress caused by a stressful event.

**FIGURE 8:**
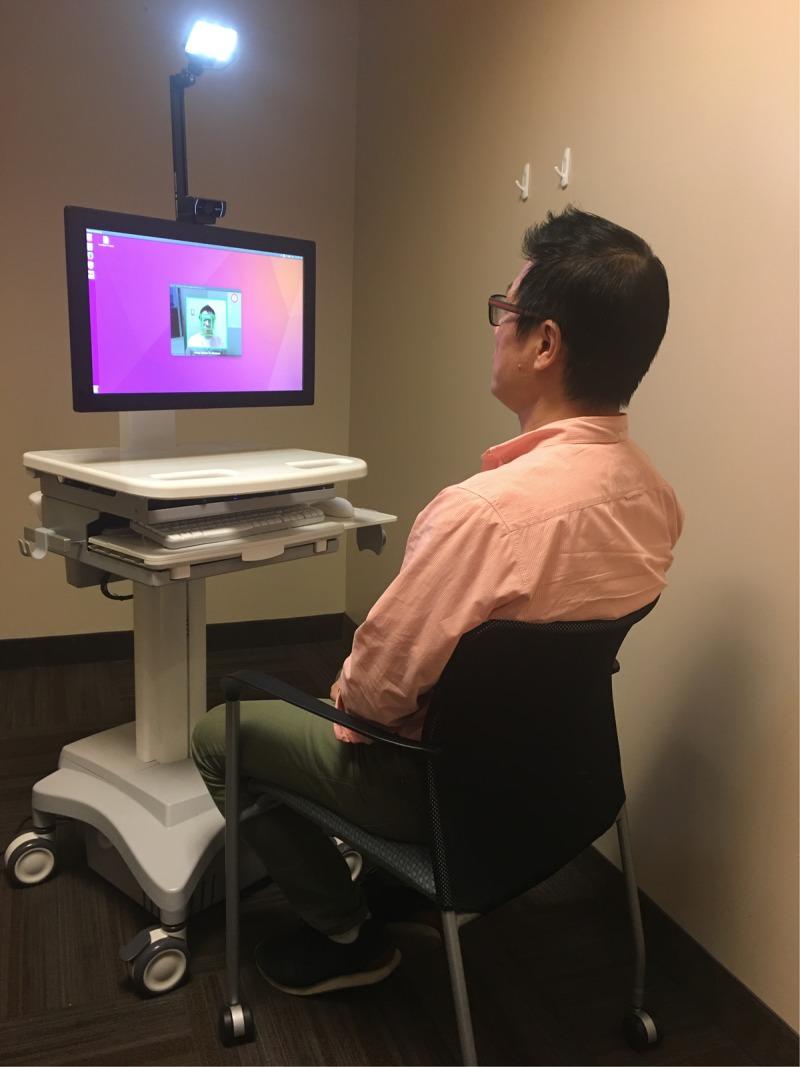
Set up of TOI station with Kang Lee, a corresponding author of this paper, sitting as a participant. All participants, whose image have been used for this paper, signed a written informed consent for release of their images in published articles prior to inclusion of their image in this paper.

Despite these limitations, the results of the present study still provide a strong proof of concept for the utility of TOI in measuring heart rate, HRV, and deriving stress measures. Given the convenient, contactless, and remotely operable features of TOI, future research would benefit in utilizing TOI to examine various aspects of stress. First, a TOI station can be placed anywhere in a research facility and used by anyone to collect data regarding a participant’s stress (**Figure 8**). This would highly benefit any study assessing stress because it would only require a small room and the click of the recording button of the webcam to determine an individual’s stress.

Second, even without a TOI station, the TOI methodology can be applied to videos captured by remotely controlled cameras. Of course such utilization of the TOI methodology can present ethical issues, potentially infringing upon personal rights to privacy. Thus, there is a need to ensure that all applications of TOI methodology to captured videos are made aware to the subject(s) of videos and consent is obtained before utilization of TOI, as was the case for the present study. With consent for video recording and application of TOI, this setup would allow for naturalistic observation of participants during stressful events as the use of a camera can be done contactlessly and remotely. This would highly benefit studies attempting to assess participants’ stress in their natural environments, during highly stressful periods (e.g., when university students are taking an exam in the examination halls) as compared to low stress periods (e.g., when the same students return from a vacation) (Melillo et al., 2011; Castaldo et al., 2016).

Third, with digital video cameras installed in mobile devices such as laptops, tablets, and mobile phones, we can use TOI at a multitude of locations (e.g., medical facilities, business offices, homes) to assess people’s stress for a long duration of time without causing discomfort. This would help medical facilities ensure the cardiovascular and psychological health of patients. It would also help organizations to ensure the efficient work of employees without endangering their health. This is particularly the case for occupations that often involve highly stressful situations (e.g., police officers, teachers, stockbrokers). Finally, TOI would help individuals to ensure their own well-being. With Apps installed in personal mobile devices such as mobile phones, we can use the existing cameras to capture video images of our face and the blood flow underneath to reflect our physiological and psychological states. This would allow people to monitor their stress anywhere they go, helping them ensure that their stress stays within a healthy range. Given that stress can lead to cardiovascular diseases, cognitive dysfunctions, and psychological disorders, the application of the TOI technology for monitoring stress would have a wide range of economic, societal, and personal benefits.

## Conclusion

The present study examined the accuracy of the TOI technology in comparison to the FDA approved BIOPAC system ECG100C for measuring heart rate and HRV, which reflects stress. We found that measurements of heart rate and SD1/SD2 (i.e., stress) obtained from the TOI technology highly corresponded with those obtained from the BIOPAC. Taken together, the findings of this study suggest that TOI can determine heart rate, HRV, and infer stress of an individual with high accuracy. Thus, the present findings reveal that the novel TOI technology can be used as a new methodology to study physiological and psychological changes in humans contactlessly, inexpensively, and conveniently.

## Author Contributions

Experimental design, data collection, experimental data analysis, and manuscript writing: JW, HL, SW, PZ, GF, and KL.

## Conflict of Interest Statement

The authors declare that the research was conducted in the absence of any commercial or financial relationships that could be construed as a potential conflict of interest.

## References

[B1] Anderson R. R. (1991). Polarized light examination and photography of the skin. *Arch. Dermatol.* 127 1000–1005. 10.1001/archderm.1991.01680060074007 2064396

[B2] Berntson G. G. Bigger J. T. Jr. Eckberg D. L. Grossman P. Kaufmann P. G. Malik M. (1997). Heart rate variability: origins, methods, and interpretive caveats. *Psychophysiology* 34 623–648. 10.1111/j.1469-8986.1997.tb02140.x 9401419

[B3] Brunsting L. A. Sheard C. (1929). The color of the skin as analyzed by spectrophotometric methods: II. The role of pigmentation. *J. Clin. Invest.* 7 575–592. 10.1172/JCI100244 16693875 PMC424596

[B4] Castaldo R. Melillo P. Bracale U. Caserta M. Triassi M. Pecchia L. (2015). Acute mental stress assessment via short term HRV analysis in healthy adults: a systematic review with meta-analysis. *Biomed. Signal Process. Control* 18 370–377. 10.1016/j.bspc.2015.02.012

[B5] Castaldo R. Xu W. Melillo P. Pecchia L. Santamaria L. James C. (2016). “Detection of mental stress due to oral academic examination via ultra-short-term HRV analysis,” in *Proceedings of the Conference Proceedings – IEEE Engineering in Medicine and Biology Society*, Honolulu, HI), 3805–3808. 10.1109/EMBC.2016.7591557 28269115

[B6] Crowley O. V. McKinley P. S. Burg M. M. Schwartz J. E. Ryff C. D. Weinstein M. (2011). The interactive effect of change in perceived stress and trait anxiety on vagal recovery from cognitive challenge. *Int. J. Psychophysiol.* 82 225–232. 10.1016/j.ijpsycho.2011.09.002 21945037 PMC3558682

[B7] Dawson J. B. Barker D. J. Ellis D. J. Grassam E. Cotterill J. A. Fisher G. W. (1980). A theoretical and experimental study of light absorption and scattering by in vivo skin. *Phys. Med. Biol.* 25 695–709. 10.1088/0031-9155/25/4/008 7454759

[B8] Demirli R. Otto P. Viswanathan R. Patwardhan S. Larkey J. (2007). *RBX Technology Overview.* Parsippany NJ: Canfields Imaging Systems.

[B9] Edwards E. A. Duntley S. Q. (1939). The pigments and color of living human skin. *Am. J. Anat.* 65 1–33. 10.1002/aja.1000650102

[B10] Friedman B. H. (2007). An autonomic flexibility-neurovisceral integration model of anxiety and cardiac vagal tone. *Biol. Psychol.* 74 185–199. 10.1016/j.biopsycho.2005.08.009 17069959

[B11] Giardino N. S. Lehrer P. M. Edelberg R. (2002). Comparison of finger plethysmograph to ECG in the measurement of heart rate variability. *Psychophysiology* 39 246–253. 10.1111/1469-8986.3920246 12212675

[B12] Jonsson P. (2007). Respiratory sinus arrhythmia as a function of state anxiety in healthy individuals. *Int. J. Psychophysiol.* 63 48–54. 10.1111/1469-8986.3920246 16989914

[B13] Kamen P. W. Krum H. Tonkin A. M. (1996). Poincaré plot of heart rate variability allows quantitative display of parasympathetic nervous activity in humans. *Clin. Sci.* 91 201–208. 10.1042/cs0910201 8795444

[B14] Kemp A. H. Quintana D. S. Gray M. A. Felmingham K. L. Brown K. Gatt J. M. (2010). Impact of depression and antidepressants treatment on heart rate variability: a review and meta-analysis. *Biol. Psychiatry* 67 1067–1074. 10.1016/j.biopsych.2009.12.012 20138254

[B15] Kofman O. Meiran N. Greenberg E. Balas M. Cohen H. (2006). Enhanced performance on executive functions associated with examination stress: evidence from task-switching and Stroop paradigms. *Cogn. Emot.* 20 577–595. 10.1080/02699930500270913

[B16] Kogan A. V. Allen J. J. Weihs K. L. (2012). Cardiac vagal control as a prospective predictor of anxiety in women diagnosed with breast cancer. *Biol. Psychol.* 90 105–111. 10.1016/j.biopsycho.2012.02.019 22414745

[B17] Lee K. Zheng P. (2016). System and method for detecting invisible human emotion. U.S. Patent No. US 2016/0098592 A1. Washington, DC: U.S. Patent and Trademark Office.

[B18] Li H. Kwong S. Yang L. Huang D. Xiao D. (2011). Hilbert-Huang transform for analysis of heart rate variability in cardiac health. *IEEE/ACM Trans. Comput. Biol. Bioinform.* 8 1557–1567. 10.1109/TCBB.2011.43 21383423

[B19] Melillo P. Bracale M. Pecchia L. (2011). Nonlinear heart rate variability features for real-life stress detection. Case study: students under stress due to university examination. *Biomed. Eng. Online* 10:96. 10.1186/1475-925X-10-96 22059697 PMC3305918

[B20] Munoz M. L. van Roon A. Riese H. Thio C. Oostenbroek E. Westrik I. (2015). Validity of (Ultra-) short recordings for heart rate variability measurements. *PLOS ONE* 10:e0138921. 10.1371/journal.pone.0138921 26414314 PMC4586373

[B21] Nishidate I. Aizu Y. Mishina H. (2004). Estimation of melanin and hemoglobin in skin tissue using multiple regression analysis aided by Monte Carlo simulation. *J. Biomed. Opt.* 9 700–710. 10.1117/1.1756918 15250756

[B22] Pan R. L. Li J. K. (2007). A noninvasive parametric evaluation of stress effects on global cardiovascular function. *Cardiovasc. Eng.* 7 74–80. 10.1007/s10558-007-9028-6 17508284

[B23] Porges S. W. (1986). “Respiratory sinus arrhythmia: physiological basis, quantitative methods, and clinical implications,” in *Cardiorespiratory and Cardiosomatic Psychophysiology*, eds Grossman P. Janssen K. Vaitl D. (New York, NY: Plenum), 101–115.

[B24] Porges S. W. (1995). Cardiac vagal tone: a physiological index of stress. *Neurosci. Behav. Rev.* 19 225–233. 10.1016/0149-7634(94)00066-A 7630578

[B25] Shaffer F. McCraty R. Zerr C. L. (2014). A healthy heart is not a metronome: an integrative review of the heart’s anatomy and heart rate variability. *Front. Psychol.* 5:1040. 10.3389/fpsyg.2014.01040 25324790 PMC4179748

[B26] Sharpley C. F. Gordon J. E. (1999). Differences between ECG and pulse when measuring heart rate, and reactivity under two physical, and two psychological stressors. *J. Behav. Med.* 22 285–301. 10.1023/A:1018724608328 10422619

[B27] Smith A.-L. Owen H. Reynolds K. J. (2013). Heart rate variability indices for very short-term (30 beat) analysis. Part I: survey and toolbox. *J. Clin. Monit. Comput.* 27 569–576. 10.1007/s10877-013-9471-4 23674071

[B28] Stamatas G. N. Zmudzka B. Z. Kollias N. Beer J. Z. (2004). Non-invasive measurements of skin pigmentation in situ. *Pigment Cell Res.* 17 618–626. 10.1111/j.1600-0749.2004.00204.x 15541019

[B29] Tavel M. E. (2001). Stress testing in cardiac evaluation: current concepts with emphasis on the ECG. *Chest* 119 907–925. 10.1378/chest.119.3.907 11243976

[B30] Tharion E. Parthasarathy S. Neelakantan N. (2009). Short-term heart rate variability measures in students during examinations. *Natl. Med. J. India* 22 63–66. 19852338

[B31] Watkins L. L. Grossman P. Krishnan R. Sherwood A. (1998). Anxiety and vagal control of heart rate. *Psychosom. Med.* 60 498–502. 10.1097/00006842-199807000-00018 9710297

